# Enhancing Rice Bran Soluble Dietary Fiber Yield Through Sequential Ultrasound–Xylanase Treatment

**DOI:** 10.3390/foods14030388

**Published:** 2025-01-24

**Authors:** Yanting Lin, Siling Zhang, Yifei Huang, Shuyuan Yang, An Zhou, Wencheng Zhang, Zeyu Wu

**Affiliations:** 1 Engineering Research Center of Bio-Process of Ministry of Education, School of Food and Biological Engineering, Hefei University of Technology, Hefei 230601, China; linyanting2021@163.com (Y.L.); slzhang2905@163.com (S.Z.); q2580779180@163.com (Y.H.); yuannasy@163.com (S.Y.); zwc1012@163.com (W.Z.); 2 The Experimental Research Center, Anhui University of Chinese Medicine, Hefei 230038, China; anzhou0901@163.com; 3 Intelligent Manufacturing Institute of HFUT, Hefei 230051, China

**Keywords:** rice bran, soluble dietary fiber, cellulase, xylanase, alternating magnetic field, ultrasound

## Abstract

The main aim of this study was to enhance the content of soluble dietary fiber (SDF) derived from rice bran (RB) through various treatments, including physical methods (ultrasound and alternating magnetic field (AMF)) and enzymatic approaches (cellulase and xylanase), applied individually or in combination. The results revealed that AMF treatment was the most effective single modification technique for increasing SDF yield, followed by treatments with xylanase, cellulase, and ultrasound. Notably, among the combined approaches, the sequential ultrasound–xylanase treatment (U-X) demonstrated the highest potential for enhancing SDF yield. Further optimization experiments revealed that under the conditions of a xylanase addition of 4.3 mg/g sample, a material-to-liquid ratio of 50 mL/g, and an ultrasonic power of 72 W, the yield of U-X-SDF significantly increased from 1.03% to 18.4%. Compared to unmodified samples, the modified SDF groups exhibited marked enhancements in water holding capacity (42.5–86.4%) and water solubility (21.0–30.6%), while the unmodified SDF displayed superior oil holding capacity than the modified groups. In summary, the sequential ultrasound–xylanase treatment not only improves the SDF yield but also enhances the functional properties of RB-derived SDF, positioning it as a valuable health-promoting food additive with potential benefits for both laboratory and industrial food applications. The optimized treatment process can contribute to the development of new functional food ingredients from RB, thereby promoting health and wellness in consumers.

## 1. Introduction

Rice, a staple food for nearly half of the global population, has been cultivated by humans for centuries [1]. Its processing generates rice bran (RB) as a major by-product. Traditionally, RB is utilized as animal feed or simply discarded [2], resulting in low utilization rates, significant environmental impacts, and wasted valuable biological resources. Consequently, there is increasing attention on the development and utilization of RB.

RB is a rich source of bioactive components, including γ-glutamic acid, ferulic acid, dietary fiber (DF), and vitamin E [3,4]. DF, composed of natural macromolecular polysaccharides, is recognized as the seventh essential nutrient group vital for human health [5]. Based on water solubility, DF is categorized into soluble dietary fiber (SDF) and insoluble dietary fiber (IDF). IDF is composed of cellulose, lignin, and insoluble hemicellulose, while SDF primarily contains pectin, gum, mucilage, and soluble hemicellulose [6]. IDF performs various physiological functions, such as increasing fecal bulk, reducing intestinal transit time, and inhibiting pancreatic lipase activity [7]. However, due to its high crude fiber content, IDF is generally not utilized in food products [8]. On the other hand, SDF exhibits a broader range of physiological activities and applications. It can be quickly metabolized by gut bacteria into short-chain fatty acids, thus modulating and enhancing the physiological functions of intestinal microorganisms [9]. Moreover, due to its high viscosity properties, SDF can increase the viscosity of chyme in the intestinal lumen, thereby prolonging gastric emptying time and slowing down small intestinal transit [10]. In addition, SDF could bind with cholesterol and sugars, leading to reduced levels of these substances within the body and minimizing their absorption and transfer in plasma. Consequently, SDF plays an important role in preventing cardiovascular disease and alleviating diabetes mellitus [11]. Upon entering the intestine, SDF also acts as a fecal softener, contributing to the prevention of constipation and hemorrhoids [12]. Furthermore, the outstanding emulsification and gel-forming capabilities of SDF ensure its great solubility in various food systems. This characteristic not only facilitates food processing but also enhances the texture and flavor of food products [13]. In consequence, SDF shows significant market potential in diverse applications, such as food additives, functional food stabilization, gelling agents, and thickeners [14]. Recently, DF derived from agricultural by-products has gained increasing recognition for its excellent physicochemical and functional properties [15]. Nevertheless, the lower content of SDF in most cereal-based DFs, compared to fruits and vegetables, seriously limits their physiological benefits and positive health impacts [16]. Given the functional and nutritional potential of RB as well as its viability as a cost-effective source of DF, there is a growing interest in exploring and developing appropriate modification methods to enhance the yield of SDF from RB.

Current methodologies for modifying DF can be broadly classified into three main approaches, i.e., physical, chemical, and biological treatments [17]. Chemical modifications often cause environmental pollution and pose potential risks to food safety [18]. These limitations have driven researchers to explore alternative methods for DF modification. Enzymatic modifications, a type of biological method, have gained widespread application in cereal processing due to their mild reaction conditions, high efficiency, and environmental sustainability [19]. Notably, cellulase and xylanase are the predominant enzymes employed in the enzymatic hydrolysis of DF. These enzymes facilitate the degradation of cellulose and hemicellulose present in DF molecules, thereby converting IDF to SDF [20]. A previous study demonstrated the efficacy of these two enzymes in increasing SDF content during the modification of RBDF [21]. Furthermore, treatments with cellulase and xylanase also positively influence the physicochemical and functional properties of DF. For example, Wang et al. [19] demonstrated that DF from cellulase-modified ginger pomace exhibited marked improvements in its water-holding capacity, swelling capacity, oil-holding capacity, and a high cation exchange capacity, cholesterol adsorption, sodium cholate binding capacity, and sodium nitrite binding capacity. Similarly, Zhu et al. [22] optimized the hydrolysis conditions of xylanase and reported a 2.23-fold increase in the cholesterol-binding capacity of xylanase-modified DF from millet bran under optimal conditions.

Recently, physical modification techniques have gained increased attention due to their potential to enhance the properties of DF. Among these techniques, ultrasonic modification has emerged as a sustainable and efficient method for altering DF properties [23]. High-intensity ultrasound has been demonstrated to effectively disrupt the crystalline structure of soybean residue fibers, thereby improving their water-holding, oil-holding, and swelling capacities [24]. These enhancements are crucial for the functionality of DF in food applications. Additionally, magnetic fields have also been widely employed in food preservation and have been shown to influence reaction kinetics by increasing collision rates between chemical substrates or accelerating diffusion rates [25]. Armenia et al. [26] reported that enzymatic reactions appeared to be activated under an alternating magnetic field (AMF). However, to the best of our knowledge, the application of magnetic field treatment for modifying DF remains scarcely explored. Moreover, the potential synergistic effects of combining different modification techniques on DF yield and quality need to be thoroughly investigated.

This study aims to explore the individual and synergistic effects of biological (cellulase and xylanase) and physical (ultrasound and AMF) modification methods on the yield and quality of rice bran soluble dietary fiber (RBSDF). Our findings will not only provide a solid theoretical foundation for the high-value utilization of RBSDF but also offer insights that could catalyze advancements in the development of novel functional foods and nutraceuticals.

## 2. Materials and Methods

### 2.1. Materials and Reagents

RB samples were kindly supplied by Xiyoumi Agricultural Technology Co., Ltd. (Hefei, China). The solutions of thermostable α-amylase (10,000 U/mL), protease (300 U/mL), and amyloglucosidase (2000 U/mL) were purchased from Lanji Biotechnology Co., Ltd. (Shanghai, China). Cellulase (10,000 U/g) and xylanase (6000 U/mg) were bought from Macklin Biochemical Technology Co., Ltd. (Shanghai, China) and Yuanye Biotechnology Co., Ltd. (Shanghai, China), respectively. All other chemicals used in this study were of analytical grade and were provided by local chemical suppliers.

### 2.2. Sample Pretreatments

RB samples were defatted using petroleum ether at a ratio of 1:25 (*w*/*v*), and this step was repeated four times. Subsequently, the defatted RB samples were dried, passed through a 60-mesh sieve, and then sealed for storage in a dryer until further use.

### 2.3. RBDF Extraction

The extraction procedure for DF was based on the enzymatic–gravimetric method [27] with slight modifications. Briefly, DF was extracted from defatted RB using distilled water at a material–liquid ratio of 1:40. The suspension underwent initial digestion with a 500 U/g α-amylase solution at 95 °C for 1 h in a shaking bath at 150 r/min. Subsequently, it was digested with a 30 U/g protease solution (pH 8.2, 60 °C, 30 min, and 150 r/min), followed by an incubation period with a 200 U/g amyloglucosidase solution (pH 4.5, 60 °C, 30 min, and 150 r/min) to remove the residual starch. Following digestion with glucoamylase, the mixture was centrifuged at 4000× *g* for 20 min, and the precipitate was dried in an oven at 60 °C to obtain IDF. A 95% ethanol (1:4 *v*/*v*) solution was added to the supernatant, and then the mixture was precipitated overnight. The residue was dried in an oven at 60 °C to obtain SDF. Finally, the IDF and SDF were combined and ground to produce rice bran dietary fiber (RBDF).

### 2.4. Single Modification of RBDF Samples

#### 2.4.1. Enzymatic Treatment

The enzymatic treatment process of RBDF samples was modified according to a previously published report [28]. Initially, RBDF was suspended in distilled water at a ratio of 1 g/40 mL (*w*/*v*) and shaken at 50 °C for 30 min to obtain a homogeneous slurry. Following pH adjustment (pH 3.5, 4.0, 4.5, 5.0, and 5.5), different amounts of cellulose (1, 5, 10, 15, and 20 mg) or xylanase (1, 2, 3, 4, and 5 mg) was added into the slurry. The mixture was then incubated in a shaking bath for a period of 1, 2, 3, 4, and 5 h. After enzyme inactivation in a boiling water bath for 20 min, the treated slurry was centrifuged at 4000× *g* for 10 min. A 160 mL of ethanol solution (950 g/kg) was added to precipitate SDF at 60 °C, and the residue was dried in an air-drying oven to obtain the enzymatically modified SDF (Cel-SDF/Xyl-SDF). The SDF yield was calculated using Equation (1).

(1) $$SDF\;yield\;\left(\%\right)=\frac{Weight\;of\;extracted\;fibre}{Sample\;weight}\times 100$$

#### 2.4.2. Ultrasound Treatment

**Figure 1:**
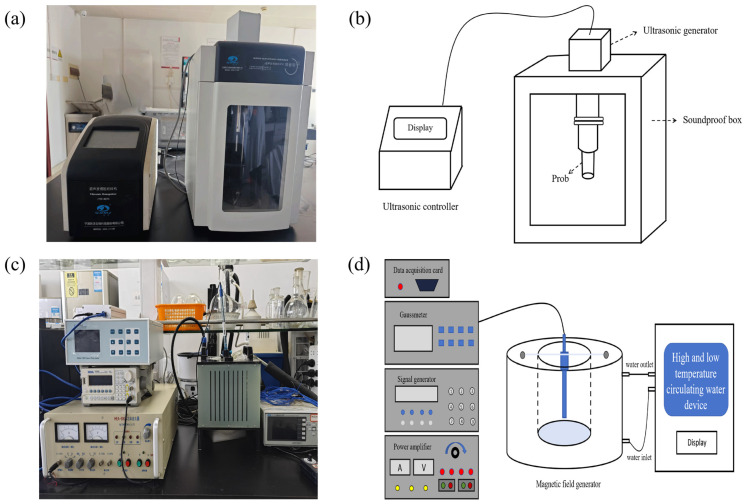
Laboratory setup and schematic diagrams of ultrasonic cell breaker (**a**,**b**) and magnetic field generation systems (**c**,**d**).

The ultrasound treatment was based on the previously proposed method [24] with slight modifications. In brief, 1 g sample was mixed with 40 mL of distilled water. The suspension was then treated with an ultrasonic cell breaker (JY98-IIIDN, Ningbo Scientz, Biotechnology Co., Ltd., Ningbo, China) equipped with a 15 mm titanium alloy probe (Figure 1a,b), and the temperature was maintained at 50 °C. Samples were subjected to different ultrasonic powers (60, 120, 240, 360, and 480 W) for specific durations (10, 20, 30, 40, and 50 min). Following the treatment, the mixture was placed in a water bath at 50 °C and shaken for a total time of 1 h. The subsequent steps of centrifugation, alcohol precipitation, and drying were carried out as described in Section 2.4.1. to obtain SDF samples after ultrasound treatment (US-SDF).

#### 2.4.3. AMF Treatment

A mixture of 40 mL distilled water and 1 g sample was placed within the magnetic field generation system (CH-Hall, Beijing Cuihai Jiacheng Magnetoelectric Technology Co., Ltd., Beijing, China, Figure 1c,d) and subjected to AMF at varying powers (2, 4, 6, 8, and 10 mT) for different durations (10, 20, 30, 40, and 50 min). Subsequent steps following magnetic field processing are detailed in Section 2.4.1 to obtain SDF samples after AMF treatment (AMF-SDF).

### 2.5. Combined Modifications of RBDF Samples

#### 2.5.1. Ultrasound-Assisted Enzyme Treatment

The simultaneous ultrasound–enzyme treatment process involved the following steps. Ultrasonic treatment was carried out according to Fan et al. [24]. After the addition of the enzyme as described in Section 2.4.1, the suspension was subjected to ultrasonic treatment. The sample was kept in an ice-water bath to remove heat and maintain the temperature at 50 °C. The ultrasonic treatment was conducted using a 20 kHz ultrasound source with different output powers (60, 120, 240, 360, and 480 W) for 10 min (3 s: 4 s work/rest cycle). Following the completion of ultrasonic treatment, the samples were placed in a 50 °C shaker for enzymatic hydrolysis, and the subsequent steps were the same as Section 2.4.1 to obtain the SDF after simultaneous ultrasound–enzyme treatment (U+C/X-SDF).

The steps of sequential ultrasound–enzyme treatment were described as follows. The RBDF suspension was first subjected to the same ultrasonic treatment as described above. Subsequently, the remaining steps were the same as those described in Section 2.4.1 to obtain the SDF after sequential ultrasound–enzyme treatment (U-C/X-SDF).

#### 2.5.2. AMF-Assisted Enzyme Treatment

The steps of simultaneous AMF–enzyme treatment were as follows. The enzyme-added samples were placed in a magnetic field generation system and treated with 6 mT AMF for 20 min. The steps after the magnetic field treatment are referred to in Section 2.4.1 to obtain the SDF after simultaneous AMF–enzyme treatment (M+C/X-SDF).

The RBDF suspension was first treated in the AMF environment as above, followed by subsequent steps as in Section 2.4.1 to obtain the SDF after sequential AMF–enzyme treatment (M-C/X-SDF).

### 2.6. Response Surface Analysis Method (RSM) Design

In accordance with the Box–Behnken design (BBD) principle, a three-factor and three-level RSM was employed to perform regression analysis and significance test on the data to determine the optimal process conditions. The coded levels and ranges of the independent variables used in the design are listed in the Supplementary Materials. Based on the outcomes of single-factor experiments, three parameters of xylanase addition (X1), liquid material ratio (X2), and ultrasonic power (X3), along with their respective range values were selected, and the SDF yield was used as the response variable for the design experiment.

The three-dimensional (3D) response surfaces, as important components of the regression equations, vividly demonstrate the interactions between two variables [29]. These visualizations elucidate the effects of experimental variable levels on the response, relationships, or interactions between two independent variables [30]. In addition, the optimal variable levels can be assessed by identifying the highest point on the 3D response surface plots. In each 3D plot, two variables were presented while the third variable was held constant at a level of zero. The curvature of the 3D plot curve indicated the significance of the experimental factors on the response value. A faster color change indicated a greater slope, which meant a more significant effect on the response value [31]. The interactions between the variables could be observed by the shape of the contour plot. An ellipse implied that the interaction between the variables had a significant effect on the response value, while a circle indicated a non-significant effect [31].

### 2.7. Structural Characterization

#### 2.7.1. Scanning Electron Microscopy (SEM)

The microstructure of the SDF powder was characterized using SEM (SU8020, Hitachi, Japan). Prior to observation, the powder samples were adhered to a double-sided carbon ribbon and subsequently coated with a thin layer of gold. Imaging was conducted at an accelerating voltage of 7 kV and a magnification of 5000×.

#### 2.7.2. Dynamic Light Scattering (DLS) Analysis

The particle size distribution of the samples was determined using a dynamic light scattering particle size analyzer (Zeta sizer Nano-ZSE, Malvern Instruments Ltd., Malvern, UK). The measurements were conducted in an aqueous medium with the SDF samples being diluted to a ratio of 1:10 [32]. The temperature was set at 25 °C, with a refractive index (RI) of 1.590 for the material and 1.330 for the dispersant.

#### 2.7.3. X-Ray Diffraction (XRD) Spectroscopy

The crystalline properties of the samples were evaluated by XRD spectroscopy. The measurements were carried out by using an X’Pert Pro MPD automated diffractometer (PANanalytical, Almelo, The Netherlands), operating at a voltage of 40 kV, and a Cu X-ray source operated at 40 mA. The recordings were made at a speed of 4°/min with a step width of 0.02° over a scanning angle (2θ) range of 5–70°. The crystallinity index (CI) was calculated according to the method described by Wang et al. [33].

(2) $$CI\;\left(\%\right)=\frac{I_{002}-I_{am}}{I_{002}}\times 100$$

where *I*_002_ was the maximum intensity of the 002 lattice diffraction peak, and *I_am_* was the diffracted intensity at 2θ = 18°.

#### 2.7.4. Fourier Transform Infrared (FT-IR) Spectroscopy

The FT-IR spectra of SDF samples were obtained using the FT-IR attenuated total reflection (ATR) mode on a Nicolet 67 FT-IR spectrometer (Thermo Scientific Inc., Waltham, MA, USA). The changes in the molecular structure of SDF samples were measured at 650–4000 cm^−1^ wavenumbers with a resolution of 4 cm^−1^.

### 2.8. Hydration Properties

#### 2.8.1. Water-Holding Capacity (WHC)

The WHC was determined using the method reported by Jiang et al. [34] with some modifications. In brief, 0.5 g of each SDF sample was mixed with 20 mL of distilled water and incubated at 37 °C for 1 h. After centrifugation at 5000× *g* for 10 min, the sediment was weighed. The hydrated residue was then dried at 105 °C to a constant weight. The WHC was defined following Equation (3).

(3) $$WHC\;(g/g)=\frac{m_{1}-m_{2}}{m_{2}}$$

where *m*_1_ and *m*_2_ were the wet and dry weights of the SDF samples, respectively.

#### 2.8.2. Oil-Holding Capacity (OHC)

The OHC was determined according to the method of Jiang et al. [34] with slight modifications. In short, 0.5 g of each SDF sample was mixed with 20 mL of soybean oil and incubated at 37 °C for 1 h. Then, the mixture was centrifuged at 5000× *g* for 20 min. The excess oil was removed, and the sediment was weighed. The calculation formula for OHC was described as follows.

(4) $$OHC\;(g/g)=\frac{m_{2}-m_{1}}{m_{1}}$$

where *m*_1_ was the weight of the SDF sample, and *m*_2_ was the weight of sediment after oil removal.

#### 2.8.3. Water Solubility (WS)

The method of Jia et al. [21] was slightly modified to determine the WS. Briefly, 0.5 g of the sample was added to 50 mL of distilled water, the mixture was stirred in a 90 °C water bath for 1 h. The supernatant was dried and weighed after centrifuging at 4000× *g* for 10 min. The WS was expressed following Equation (5).

(5) $$WHC\;(g/g)=\frac{m_{1}}{m_{2}}$$

where *m*_1_ was the remaining weight of the supernatant after drying, and *m*_2_ was the weight of the SDF sample.

### 2.9. Statistical Analysis

All experiments were performed in triplicate, and the results were expressed as mean ± standard deviation (SD). Design-Expert 13.0 software, SPSS software, Origin 9.0, and Graphpad Prism 10 software were utilized for response surface testing, statistical analysis, and graphing, respectively. A significance level of *p* < 0.05 was considered statistically significant.

## 3. Results and Discussion

### 3.1. Effects of Different Treatments on SDF Yield

**Figure 2:**
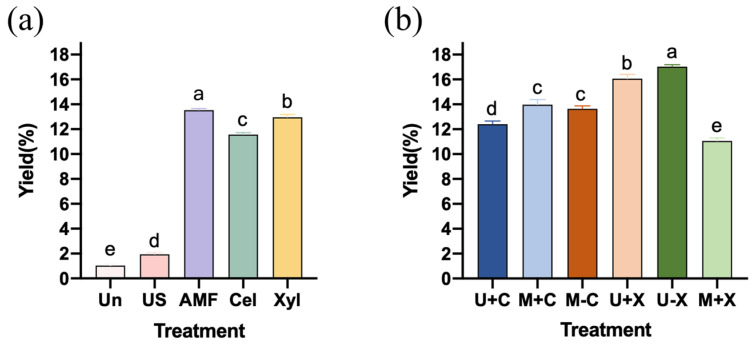
Effects of single modification (**a**) and combined modification (**b**) on the SDF yield. Note: There was a significant difference (*p* < 0.05) between different lowercase letters.

The impact of diverse treatment methods on the SDF yield is illustrated in Figure 2. As shown in Figure 2a, compared to unmodified SDF samples (Un, 1.03%), all four individual treatments significantly increased the SDF yield (*p* < 0.05), with the following order: AMF (13.53%) > Xyl (12.97%) > Cel (11.57%) > US (1.93%). This augmentation could be attributed to the enzymatic hydrolysis by cellulase and xylanase, which target cellulose and hemicellulose fractions within IDF, thereby raising the conversion to SDF. These observations were consistent with previous studies conducted by Rivas et al. [35] and Zhou et al. [36]. Simultaneously, ultrasound and AMF treatments disrupted the cell wall structure of RBDF, facilitating the transition from IDF to SDF and enhancing SDF release, which led to a higher production of SDF [37]. However, the increase in SDF yield of ultrasound-modified samples was markedly lower than that of enzyme- and AMF-modified groups, implying that enzyme and AMF strategies were more effective in improving the SDF/IDF ratio than the ultrasound approach. These findings suggested the promising potential of AMF as a novel and high-efficient technique for DF modification.

Figure 2b illustrates the effects of combined enzymatic and physical modifications on the SDF yield. To summarize, all five combined modification methods led to significantly higher SDF yields compared to their corresponding single treatments (*p* < 0.05), in the following order: U-X (17.03%) > U+X (16.07%) > M+C (13.97%) > M-C (13.63%) > U+C (12.47%). However, the M+X treatment (11.07%) proved less effective than either xylanase or AMF treatments alone, indicating that not all combined modifications enhanced the efficacy over single treatments. The SDF yield achieved through the synergistic effect of ultrasound and enzymatic treatments was significantly higher than that of US-SDF or Cel/Xyl-SDF (*p* < 0.05). Notably, the sequential application of ultrasound followed by xylanase treatment (U-X) was significantly more effective than simultaneous ultrasound–xylanase treatment (U+X, *p* < 0.05), likely due to reduced energy requirements. This superiority could be attributed to ultrasonic pretreatment enhancing the accessibility of DF for subsequent enzymatic hydrolysis, thereby increasing efficiency. Enzyme activity was found to be modulated by varying physical fields. Specifically, cellulase exhibited its highest SDF yield under an ultrasonic field, whereas xylanase demonstrated optimal performance in an AMF environment. As depicted in Figure 2b, neither sequential nor simultaneous modifications altered the efficacy of cellulase when assisted by AMF. Compared to Cel-SDF, M+C-SDF showed a significant increase in yield (*p* < 0.05), remaining much lower than AMF-SDF. Conversely, the yield of M+X-SDF was even less than that of Xyl-SDF or AMF-SDF. These findings suggested that the choice and sequence of physical treatments could significantly influence enzymatic modifications, ultimately affecting the yield and characteristics of the extracted SDF. Zhang et al. [25] reported the enhancement of cellulase activity by AMF. However, as far as we know, research on the effect of magnetic field treatment on xylanase activity is scarce. Our findings prompt the hypothesis that AMF may have an inhibitory effect on xylanase activity. In conclusion, AMF was a promising physical modification method for single treatments, while the sequential ultrasound–xylanase treatment emerged as the preferred strategy for combined modifications.

### 3.2. Optimization of Modification Conditions

The experimental conditions for the U-X treatments were optimized utilizing RSM, with the SDF yield as the evaluation index. Based on the results obtained from single-factor experiments, the factors and levels for BBD were determined and are present in Table 1. Among the coded variables, the quadratic model fitted for SDF yield can be explained by the following quadratic regression equation.

Y = 18.03 + 0.975A − 0.2375B + 0.1625C + 0.1AB + 0.25AC − 0.125BC − 0.9792A^2^ − 0.6542B^2^ − 0.8542C^2^

where Y was the SDF yield (%), and A, B, and C were the coded variables.

Analysis of variance (ANOVA) was employed to assess and screen the effects of significant variables in both linear and quadratic forms. The results presented in Table 2 indicated that the *p*-value for the quadratic model was 0.0002, suggesting that the model was highly significant. Additionally, the lack-of-fit was not significant (*p* = 0.0642), which meant that the regression model fitted well, and the test error was sufficiently small to confidently analyze the results in place of the true values [38]. The R^2^ value of 0.9901 and the AdjR^2^ value of 0.9723 demonstrated that the model had a high level of reliability and reflected the changes in the response values [39]. Specifically, factors A, B, C, AC, A^2^, B^2^, and C^2^ were all found to be significant (*p* < 0.05), revealing that xylanase addition, liquid-to-feed ratio, and ultrasonic power were all significantly correlated with SDF yield.

**Figure 3:**
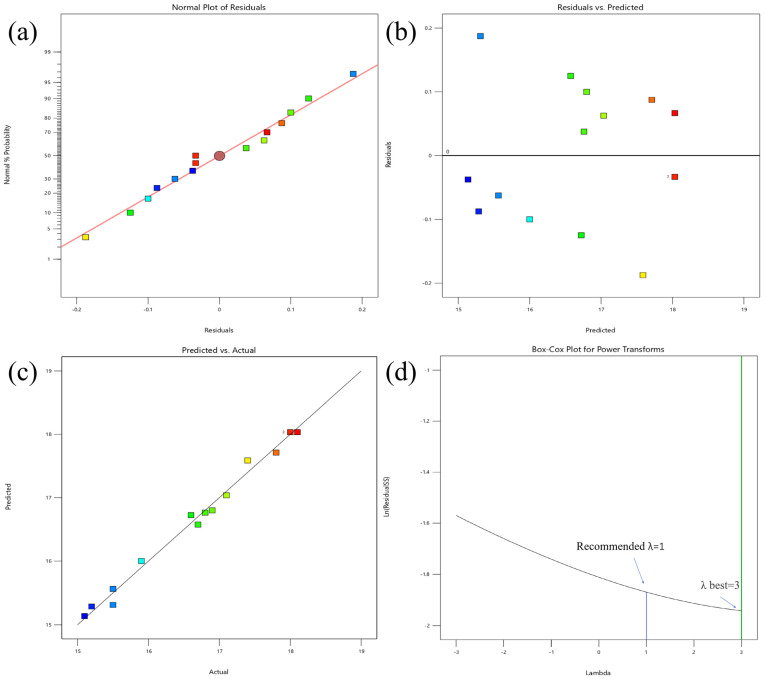
Residual normal probability (**a**), residual vs. predicted value (**b**), actual vs. predicted value (**c**), and Box–Cox (**d**) plots for the model.

Diagnostic and correlation plots played a crucial role in evaluating the significance of ANOVA outcomes [40]. To further validate the accuracy of the optimized design, both a normal plot of residuals and a Box–Cox plot for power transformation were generated. Figure 3 presents four key visualizations, that is, (a) the normal plot of residuals, (b) the residuals vs. predicted values plot, (c) the actual vs. predicted values plot, and (d) the Box–Cox plot for the model. The results revealed that the plotted residuals followed an approximate normal distribution and were irregularly scattered relative to the predicted values. Additionally, a near-linear relationship was observed between the actual and predicted values, suggesting a well-fitted response surface model. The Box–Cox plot was employed to determine the most appropriate power law transformation [40]. The lowest point of the Box–Cox plot showed the optimal value of lambda (λ), which indicated the minimized residual sum of squares. Response surface models exhibited the lowest point in the Box–Cox plot, demonstrating the transfer ability of the model [41].

**Figure 4:**
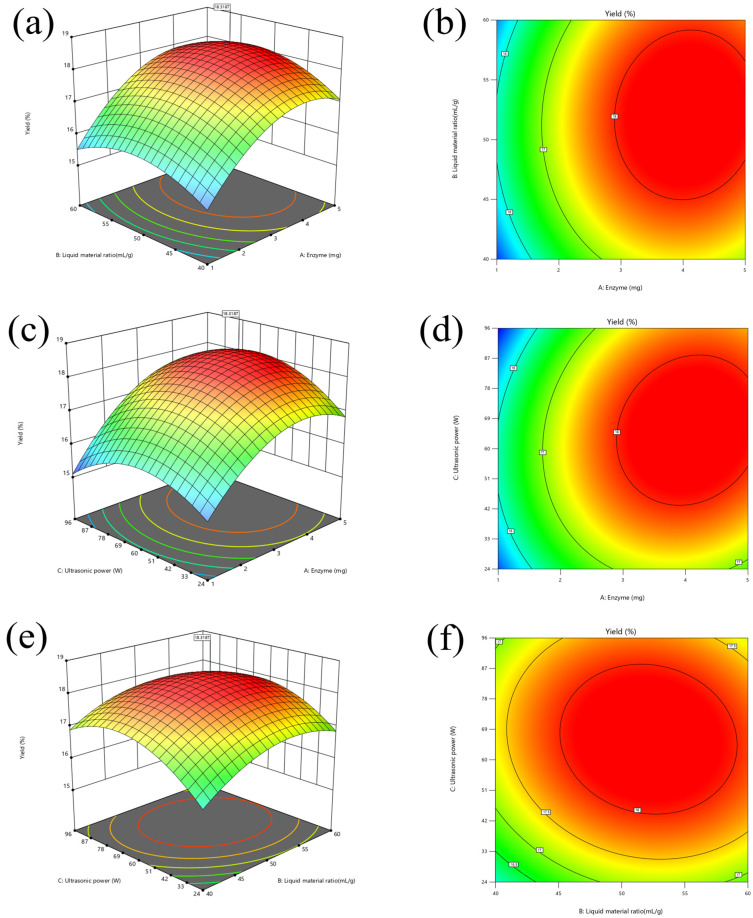
Response surface plots for Y = f (A, B) (**a**,**b**), Y = f (A, C) (**c**,**d**), and Y = f (B, C) (**e**,**f**).

Figure 4 presents the 3D response surfaces and contour plots generated by the model. Figure 4e,f showed a weak interaction between liquid material ratio (B) and ultrasonic power (C). In contrast, a robust interaction existed between xylanase addition (A) and ultrasonic power (C), as depicted in Figure 4c,d.

The numerical optimization results revealed that the optimal conditions for SDF production were a xylanase addition of 4.288 mg/g sample, material–liquid ratio of 48.921 mL/g, and ultrasonic power of 71.844 W, predicting a maximum yield of 18.23%. However, considering practical feasibility, these were adjusted to a xylanase addition of 4.3 mg, material-liquid ratio of 50 mL/g, and ultrasonic power of 72 W. Under these optimized conditions, three validation experiments were conducted, resulting in an average U-X-SDF yield of 18.4%, which was closely consistent with the predicted value. This confirmed the reliability and accuracy of the response surface model.

### 3.3. SEM Analysis

**Figure 5:**
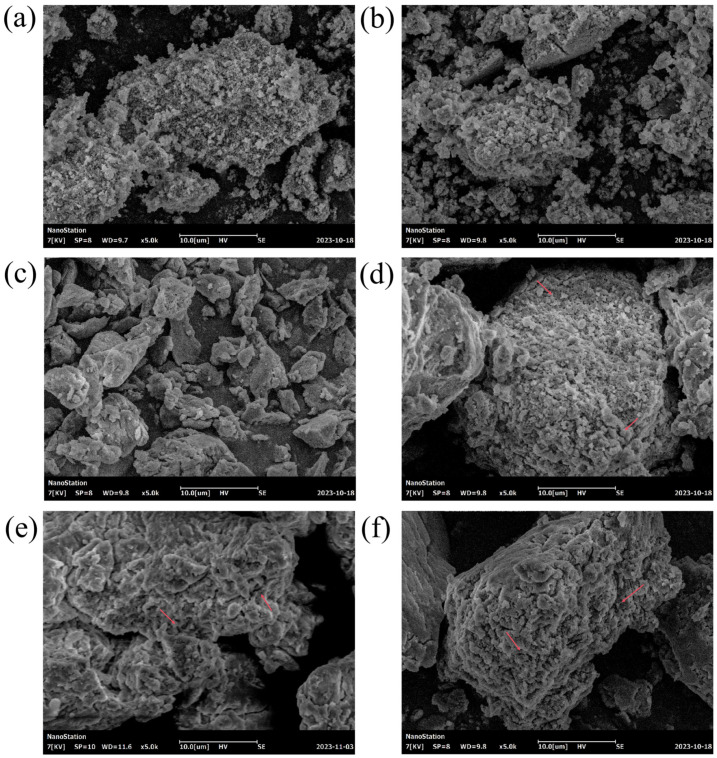
SEM micrographs of different SDF samples. Un-SDF (**a**), Cel-SDF (**b**), AMF-SDF (**c**), M+C-SDF (**d**), Xyl-SDF (**e**), and U-X-SDF (**f**).

The morphological changes of SDF samples under different treatments are shown in Figure 5. The SEM images demonstrated that the Un-SDF samples had a dense and compact structure with small irregular particles and no obvious fragments (Figure 5a). The destruction of SDF samples was weaker because of the milder reaction conditions of the enzymatic method [42]. In contrast, the samples treated with cellulase (Figure 5b) and xylanase (Figure 5e) displayed an increased number of pores and cracks on their surfaces, although their overall microstructure remained largely intact. This was because the enzymatic hydrolysis process broke off certain glycosidolysis, leading to these structural alterations [43]. Comparing Figure 5b and Figure 5e, it was evident that the porosity of the samples treated with xylanase (Figure 5e) surpassed that of the cellulase-treated samples (Figure 5b). This suggested that xylanase possessed a more potent hydrolytic capability than cellulase, which was consistent with the higher yield of xylanase-treated SDF compared to cellulase-treated SDF.

In contrast, the structure of SDF samples after AMF, AMF-assisted cellulase, and ultrasound-assisted xylanase treatments exhibited dramatic changes. The surface of AMF-treated SDF samples appeared relatively dense and smooth, with the structure breaking into numerous fragments and reduced particle size under the magnetic field environment (Figure 5c). The high SDF yield obtained by AMF modification could be attributed to its severely fragmented structure, which facilitates better dissolution of SDF. The surfaces of C+M-SDF (Figure 5d) and U-X-SDF (Figure 5f) samples were characterized by extensive cracks and pores, presenting cellular network structures with enhanced porosity. This was because of the cavitation effects of ultrasound and the disruptive effects of the magnetic field on the fiber structure, leading to the cross-linking breakage of polysaccharide molecules [44]. Cellulose, hemicellulose, and lignin are typically present as polymers within the cell wall [10]. As external physical forces disrupted the fiber structure, the interconnections between these components were broken, allowing more enzymes to penetrate the interior of the fiber for hydrolysis. Consequently, the modified SDF samples exhibited a looser and more porous structure [45]. The microstructure of SDF samples played a crucial role in determining their hydration characteristics [11,46]. A loose and porous structure increases the specific surface area and exposes more functional groups, thereby promoting the capacity for water adsorption and binding [47]. These characteristics have significant implications for the application of modified SDF in food products, potentially improving water retention and textural qualities.

### 3.4. Particle Size Analysis

Table 3 indicated that the particle size of modified SDF samples was significantly reduced (*p* < 0.05). Specifically, the particle sizes of the SDF samples treated with Cel, M+C, Xyl, AMF, and U-X sequentially decreased. However, there was no significant difference among the three treatment groups of Cel, M+C, and Xyl (*p* > 0.05). The above results were consistent with the images presented by SEM.

### 3.5. XRD Analysis

**Figure 6:**
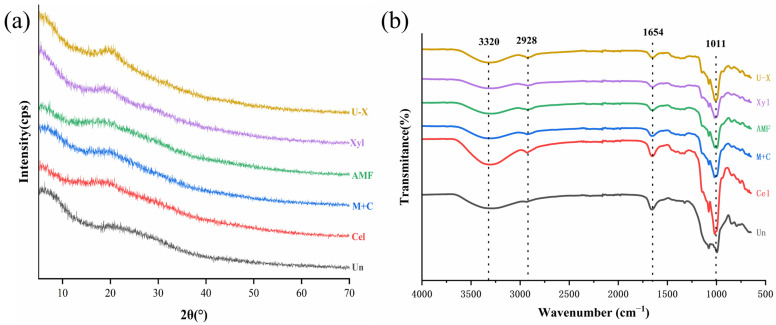
XRD patterns (**a**) and FTIR spectra (**b**) of RBSDF samples before and after modification.

The corresponding XRD spectra and CI of different modified SDF samples are shown in Figure 6a and Table 3, respectively. The various modification treatments affected the crystal structures and CI of the SDF samples. As depicted in Figure 6a, each treatment group exhibited a smooth and broad diffraction peak near 20°, characteristic of the typical cellulose I-type crystal structure [47]. The modified treatment groups displayed similar characteristic peaks, with variations only in peak intensity, indicating no change in the crystalline shape of SDF before and after modification. However, the CI of the modified SDF samples decreased by 5.56–16.54% compared to Un-SDF (Table 3), suggesting a reduction in the ordering of the crystal structure [44]. This was attributed to an increased content of amorphous components due to enzymatic, ultrasonic, and AMF treatments, leading to a higher proportion of amorphous regions [10]. In addition, the CI of the physically modified SDF decreased more significantly. This change may be related to the mechanical and cavitation effects that reduced the particle size of SDF, potentially causing some degradation of cellulose and hemicellulose [13]. The polysaccharide chains in the crystalline region are interconnected by strong inter- and intramolecular hydrogen bonds, forming a highly ordered structure. This led to significant resistance to enzyme or chemical reactions with the active substances within the SDF [10]. A previous study reported that a decrease in crystallinity suggested a reduction in the polymerization degree of SDF and a looser structure, which could influence its physicochemical properties [33].

### 3.6. FT-IR Analysis

Figure 6b illustrates the FT-IR spectra for both modified and unmodified RBSDF samples. The spectral patterns across all SDF samples were fundamentally analogous, with variations primarily observed in the absorption intensity of specific characteristic peaks. All SDF groups exhibited a broad absorption band around 3320 cm^−1^, which was characteristic of the O-H stretching vibrations associated with hydrogen bonds [48,49]. In comparison to the unmodified samples, all modified samples, except for Cel-SDF, showed a weaker peak at the 3320 cm^−1^ band, indicating the breaking of intramolecular hydrogen bonds within cellulose and hemicellulose [50]. This broad band also implied the presence of pectin and hemicellulose [24]. A small peak at 2928 cm^−1^ was attributed to C-H antisymmetric stretching and bending vibrations in the polysaccharide methylene groups, representing polysaccharide compound structures [44]. The distinct sharp peak at 1654 cm^−1^ corresponded to the characteristic absorption peak of C=O in uronic acid [51,52,53], with cellulase treatment increasing the uronic acid content in SDF samples. The stronger absorption peaks at 1011 cm^−1^ corresponded to the vibration, bending, and stretching of lignin or hemicellulose ether bonds (C-O-C) [15]. After modification treatments on RBDF, there was an obvious decrease in the intensity of these peaks, implying partial degradation of hemicellulose [54] and the conversion of DF into oligosaccharides [46]. Overall, biological and physical modifications destroyed the intermolecular hydrogen bonds of cellulose and hemicellulose and caused degradation of the polymers [14]. Moreover, the applied treatments appeared to increase hydrophilic groups and water-binding sites [55].

### 3.7. WHC, OHC, and WS Analysis

The hydration characteristics of RBSDF samples, both pre- and post-modifications, are present in Table 4. Herein, WHC refers to the capacity of these samples to retain water against external centrifugal forces [21]. A high WHC in DF can prevent food from shrinking due to the dehydration process and modulate food viscosity [56], thereby exerting a favorable influence on food quality. As observed in Table 4, all modified SDF samples exhibited significantly higher WHC values (*p* < 0.05) than those of Un-SDF samples. Notably, Xyl-SDF had the highest WHC value, representing a 1.86-fold increase compared to Un-SDF samples. This enhancement of WHC could be attributed to several factors, including the reduction in fiber particle size [44], the increase in porosity and specific surface area [57], and the sparse microstructure of SDF samples. The latter change facilitated greater exposure of hydrophilic groups [14], further enhancing water retention capabilities.

WS reflects the degree of SDF dissolved by water at a certain temperature [47]. WS is positively correlated with SDF content [58], making it a crucial reference index for SDF. Combined with the results in Table 4 as well as Figure 2 and Figure 4, the content of WS corresponded to the content of SDF. Among all the modified treatment groups, the largest values of both WS and SDF were found in the U-X groups.

OHC serves as an important indicator for assessing the retention of fats and fat-soluble flavor compounds during food processing, so SDF is also used as a fat substitute [51]. The OHC of Cel-SDF did not exhibit a statistically significant deviation (*p* > 0.05) from that of Un-SDF. However, the OHC values of the other modified SDF samples were significantly lower (*p* < 0.05). The above results could be elucidated through dual perspectives. Firstly, the modification process likely facilitated the exposure of hydrophilic groups within the SDF matrix, thereby weakening the hydrophobicity and leading to a decrease in OHC [42]. Secondly, a positive correlation between lignin content and OHC value has been documented [56]. Supporting this, FT-IR analyses revealed that lignin content was associated with an absorption peak at 1011 cm^−1^. Notably, the intensity of this peak was much lower in SDF samples from groups exhibiting reduced OHC, compared to the unmodified and cellulase-treated groups (Figure 6b), suggesting the potential role of lignin in modulating OHC.

## 4. Conclusions

In this study, we explored various modification techniques to enhance the yield of RBSDF. Among these methods, AMF, as a novel, safe, and efficient physical approach, proved to be the most effective single modification method, significantly increasing the RBSDF yield from 1.03% ± 0.12 to 13.53% ± 0.12. When combining different techniques, the sequential application of ultrasound followed by xylanase hydrolysis produced the highest RBSDF yield in this study, reaching up to 18.4% under optimal conditions of a xylanase addition of 4.3 mg/g sample, the feed/liquid ratio of 50 mL/g, and the ultrasound power of 72 W. SEM, DLS, and FT-IR analyses revealed that the modified RBSDF had a looser structure with increased porosity and reduced particle size, accompanied by changes in the intensity of functional group absorption peaks. In addition, all modified SDF samples exhibited better performance in WHC and WS, but lacked in OHC. However, the practical application of modified RBDF is still in the laboratory research phase. To integrate it into actual factory environments, except for considering its performance characteristics, it is also extremely important to comprehensively assess issues related to feasibility, operational costs, and heat energy consumption during the practical operation process. Overall, the use of ultrasound, AMF, and enzyme treatments effectively enhanced the physicochemical properties and structural characteristics of RBSDF, thereby improving its potential as a functional food additive and increasing the comprehensive utilization value of RB.

## Figures and Tables

**Table 1:** BBD with observed responses and predicted values for the SDF yield.

Run	X1	X2	X3	Yield
1	1	50	96	15.1
2	1	60	60	15.5
3	3	60	96	16.9
4	3	40	96	16.7
5	3	50	60	18.0
6	5	60	60	17.8
7	3	40	24	15.9
8	1	50	24	15.5
9	1	40	60	15.2
10	5	50	96	17.4
11	5	50	24	16.8
12	3	50	60	18.1
13	5	40	60	17.1
14	3	60	24	16.6
15	3	50	60	18.0

**Table 2:** ANOVA for the response surface quadratic model.

Source	Sum of Squares	df	Mean Square	F-Value	*p*-Value	Significance
Model	15.44	9	1.72	55.62	0.0002	**
A-enzyme	7.6	1	7.6	246.65	<0.0001	**
B-liquid material ratio	0.4512	1	0.4512	14.64	0.0123	*
C-ultrasonic power	0.2112	1	0.2112	6.85	0.0472	*
AB	0.04	1	0.04	1.3	0.3063	
AC	0.25	1	0.25	8.11	0.0359	*
BC	0.0625	1	0.0625	2.03	0.2138	
A^2^	3.54	1	3.54	114.81	0.0001	**
B^2^	1.58	1	1.58	51.25	0.0008	**
C^2^	2.69	1	2.69	87.37	0.0002	**
Residual	0.1542	5	0.0308			
Lack of Fit	0.1475	3	0.0492	14.75	0.0642	not significant
Pure Error	0.0067	2	0.0033			
Cor Total	15.59	14				

Significance at * *p* < 0.05 and at ** *p* < 0.01.

**Table 3:** Average particle size and crystallinity index of SDF samples.

	Un-SDF	Cel-SDF	C+M-SDF	AMF-SDF	Xyl-SDF	U-X-SDF
Z-average size (d.nm)	879.8 ± 61.79 ^a^	627.9 ± 17.67 ^b^	590.2 ± 78.32 ^b^	459.2 ± 37.65 ^c^	559.4 ± 48.84 ^b^	342.8 ± 44.7 ^d^
CI	18.50%	7.43%	5.82%	1.96%	12.94%	4.39%

Data are expressed by means ± standard deviation. Values with different letters in the same column are significantly different (*p* < 0.05).

**Table 4:** Hydration properties of SDF samples.

	Un-SDF	Cel-SDF	C+M-SDF	AMF-SDF	Xyl-SDF	U-X-SDF
WHC (g/g)	2.28 ± 0.31 ^c^	3.29 ± 0.35 ^b^	4.20 ± 0.5 ^a^	3.85 ± 0.35 ^ab^	4.25 ± 0.59 ^a^	3.25 ± 0.46 ^b^
WS (g/g)	0.62 ± 0.04 ^b^	0.68 ± 0.03 ^b^	0.75 ± 0.06 ^a^	0.78 ± 0.03 ^a^	0.76 ± 0.02 ^a^	0.81 ± 0.06 ^a^
OHC (g/g)	1.64 ± 0.14 ^a^	1.44 ± 0.19 ^ab^	1.15 ± 0.11 ^c^	1.19 ± 0.13 ^bc^	1.02 ± 0.13 ^c^	1.17 ± 0.16 ^c^

Data are expressed by means ± standard deviation. Values with different letters in the same column are significantly different (*p* < 0.05).

## Data Availability

The original contributions presented in this study are included in the article/Supplementary Materials. Further inquiries can be directed to the corresponding author.

## Supplementary Materials

The following supporting information can be downloaded at: https://www.mdpi.com/article/10.3390/foods14030388/s1. Table S1: Factors and levels in the response surface analysis.
