# Supplementary material for: Enhancing Rice Bran Soluble Dietary Fiber Yield Through Sequential Ultrasound–Xylanase Treatment
Source: Foods. 2025 Jan 24;14(3):388. doi: 10.3390/foods14030388 (PMC11817479; doi:10.3390/foods14030388)
Supplement: Supplementary file 1 [file foods-14-00388-s001.zip › foods-3326164-supplementary.pdf]

Table S1 Factors and levels in the response surface analysis.

Independent Variables	Symbol		Levels		
	Un-Coded	Coded	−1	0	1
Xylanase addition (mg)	X1	X1	1	3	5
Liquid material ratio (mL/g)	X2	X2	40	50	60
Ultrasonic power (W)	X3	X3	24	60	96
